# Resource Management in Cloud Radio Access Network: Conventional and New Approaches

**DOI:** 10.3390/s20092708

**Published:** 2020-05-09

**Authors:** Rehenuma Tasnim Rodoshi, Taewoon Kim, Wooyeol Choi

**Affiliations:** 1 Department of Computer Engineering, Chosun University, Gwangju 61452, Korea; r.rodoshi25@gmail.com; 2 School of Software, Hallym University, Chuncheon 24252, Korea; taewoon@hallym.ac.kr

**Keywords:** cloud radio access network, resource management, RRM, CRM, RRH clustering

## Abstract

Cloud radio access network (C-RAN) is a promising mobile wireless sensor network architecture to address the challenges of ever-increasing mobile data traffic and network costs. C-RAN is a practical solution to the strict energy-constrained wireless sensor nodes, often found in Internet of Things (IoT) applications. Although this architecture can provide energy efficiency and reduce cost, it is a challenging task in C-RAN to utilize the resources efficiently, considering the dynamic real-time environment. Several research works have proposed different methodologies for effective resource management in C-RAN. This study performs a comprehensive survey on the state-of-the-art resource management techniques that have been proposed recently for this architecture. The resource management techniques are categorized into computational resource management (CRM) and radio resource management (RRM) techniques. Then both of the techniques are further classified and analyzed based on the strategies used in the studies. Remote radio head (RRH) clustering schemes used in CRM techniques are discussed extensively. In this research work, the investigated performance metrics and their validation techniques are critically analyzed. Moreover, other important challenges and open research issues for efficient resource management in C-RAN are highlighted to provide future research direction.

## 1. Introduction

Advances in Internet of Things (IoT) technology have increased the number and usage of wireless sensor nodes [1,2]. The rapidly increasing smart devices and IoT modules in volume generate an enormous amount of data traffic, putting an additional burden on existing mobile wireless sensor networks. According to Cisco, the overall mobile data traffic is forecasted to increase to 77 exabytes per month by 2022, which is a seven-fold increase over that in 2017 [3]. Moreover, the average traffic generated by smartphones will be 11 GB per month, more than a four and half-fold increase over that in 2017. To tackle this enormous data traffic, the capacity of traditional mobile network architectures and the available resources are not sufficient. Therefore, mobile network operators (MNOs) need to increase the number of active base stations to satisfy the increasing user demand. To achieve this, the MNOs would be required to incur exceptionally high operational expenditures (OPEX) and capital expenditures (CAPEX) for deploying more base stations or maintaining and operating the existing stations [4]. Traditional radio access networks (RANs) would become exceptionally expensive if they are to remain competitive in the future mobile internet world. The deployment of more base stations would also increase the power and energy consumption [5]. Furthermore, owing to the dynamicity of the user traffic load, some base stations would be overloaded, whereas others would not be used fully. This would result in a low utilization rate and an inefficient use of base stations.

One of the novel network architectures that can potentially address the above-mentioned challenges is C-RAN architecture [4,5]. The key concept of C-RAN is to break down the base station functionalities by decoupling the base station into Base band unit (BBU) and Remote radio head (RRH), and then centralizing the BBUs from multiple sites into a single geographical point such as a cloud data center, using cloud computing and virtualization techniques. BBUs are responsible for baseband signal processing, whereas RRHs take responsibility for signal amplification and modulation. RRHs are deployed with an antenna at the cell site and are connected to the BBU pool with a fronthaul link. As the baseband processing is centralized in a virtualized BBU pool, it can adapt to non-uniform traffic and utilize the resources effectively. Many operators can share the same BBU pool by renting it as a cloud service. Moreover, the network performance is improved by reducing the delay caused by handover. The operational cost can be reduced because the BBUs are centralized at a single site. Furthermore, the addition and upgrade of a new BBU is more convenient compared to a traditional RAN. MNOs only need to install an RRH to increase the network capacity. It also reduces the power and energy consumption, which eventually decreases the network costs. 

Although C-RAN appears to be a promising architecture for ensuring low cost and energy efficiency, a few challenges need to be resolved for maximizing its advantages. The co-location of many BBUs in a BBU pool connected to RRHs requires a reliable link with a high bandwidth capacity. The BBU–RRH association needs to be done in a way so that the BBU resources are utilized effectively, and user demands are also fulfilled. Effective strategies for base station mode switching must be adopted considering the real-time environment. 

The efficient management of the resources in C-RAN to satisfy user demand is a significant challenge due to the user mobility and dynamic environment. The resource in wireless communication mainly refers to the radio frequency (RF) spectrum, which is very limited. For optimal resource management, the RF resource must be utilized effectively by maximizing the number of users getting service. The objective of resource management is to utilize the limited radio frequency spectrum resources and radio network infrastructure with maximum efficiency. Different resource allocation mechanisms have been proposed for efficient resource management in C-RAN. Resource management techniques could be static or dynamic depending on whether the environment is static or dynamic. In the latter case, the dynamicity of traffic load, user positions, user mobility, QoS requirement, and base station density are considered. Different RRM strategies for improving spectral efficiency have been proposed, such as efficient static or dynamic channel allocation, transmit power control, spectrum management, cache management, link adaptation, user association, beamforming, and handover criteria.

### 1.1. Review of Existing Surveys

Many surveys have been performed considering resource allocation and scheduling in cloud [6], resource sharing in heterogeneous cloud radio access network (H-CRAN) [7], resource management in 5G RAN [8], energy-efficient base station switching techniques in green cellular network [9], energy-efficient wireless communication [10], radio resource management in machine-to-machine communication [11], strategies for switching off base stations in heterogeneous networks [12], and clustering techniques for RRHs [13]. The topics of the previous surveys along with their respective publishing years, and the areas they covered in those surveys are listed in Table 1. 

The authors in [6,14,15,16,17,18] presented surveys through investigating various works related to resource management methods in a cloud computing environment. Guzek et al. discussed the studies on the application of computational intelligence tools in cloud computing resource optimization problems by dividing the resource management problems into static and dynamic problems [15]. This work further identified a few alternative methods and unexplored territories with regard to the optimization of cloud resource management, for future researchers. A short survey regarding the resource allocation and scheduling methods for application in the cloud is presented in [6]. Jennings et al. outlined a conceptual framework for cloud resource management and used it to structure the state-of-the-art review; furthermore, they identified a few challenges for future investigation based on the review [14]. Singh et al. presented a methodical analysis of resource scheduling in cloud computing, resource scheduling algorithms, and resource management [16]. The study also analyzed the types and benefits of resource management tools as well as resource scheduling aspects and resource allocation policies. The article further recommended a few research directions for future works. Mohammadiah et al. studied the research issues in the area of resource management, specifically, resource allocation and monitoring in the cloud-computing environment. They also studied solution approaches [17]. Demicri et al. presented a comparative study of the works that employed machine learning to offer solutions for energy efficiency in cloud computing environments along with some future research directions [19]. 

The related works on resource management techniques for H-CRANs have been reviewed in [7,12,20,21,22]. In [7], the resource sharing opportunities in H-CRAN were investigated considering the spectrum, infrastructure, and network levels. The major challenges in deploying dynamic resource sharing in this architecture and the research direction for addressing these challenges were also discussed. In addition, a few trending technologies that are essential for resource sharing in an H-CRAN were highlighted. Finally, a simulation of spectrum and infrastructure sharing in the H-CRAN environment was presented with results. Meanwhile, in [20], a comprehensive review of the works on the energy efficiency of H-CRAN and cloud computing was presented individually in conjunction with a discussion of the challenges and continuing issues in joint deployment. The authors discussed the energy efficiency issue for each building block of the network architecture. The authors in [21] reviewed the recent advances in HetNets, in conjunction with the architecture evolution, key techniques, and continuing research issues. They presented the system architectures of traditional HetNets, H-CRANs, and F-RANs (fog radio access networks). Moreover, a novel performance metric called economic energy efficiency was introduced with self-organizing HetNets and access slicing techniques. In addition, the key techniques across the physical, MAC, and network layers were elaborated. In [22], a comprehensive assessment was carried out to compare the existing radio resource management schemes proposed for LTE/LTE-A femtocell and relay networks, in terms of interference mitigation, radio resource utilization, fairness, complexity, and QoS. The authors also identified future challenges and potential research directions for RRM development and HetNet enhancement. The authors in [12] reviewed the works on designing base station switching-off strategies from different design perspectives such as random; distance-aware; load-aware; auction-based; and joint design with user association, resource allocation, and physical-layer interference cancellation strategies. 

Many research works have been proposed considering the 5G mobile communication systems. Furthermore, a few surveys have been conducted discussing the works on 5G networks, such as [8,19,23,24]. The authors in [6] presented a comprehensive survey on radio interference and resource management schemes for 5G RAN. They classified the schemes into radio interference, energy-efficient, spectrum efficient, and hybrid resource management. They further made a few recommendations on the continuing research issues of 5G RAN systems that were deduced from the schemes reviewed. Meanwhile, a survey [17] was conducted recently on the research studies on C-RAN focusing on throughput enhancement, interference management, energy efficiency, latency, security, and system cost reduction for 5G cellular systems. The authors in [21] presented a comprehensive review of the revolution of RAN architectures, wherein they mentioned the architectures of C-RAN, H-CRAN, virtualized C-RAN, and F-RAN. Moreover, a comparative analysis based on the energy consumption, security, CAPEX/OPEX, performance, spectrum, mobility, resource allocation, and system architectures for fulfilling user requirements, was presented. In [24], a review of state-of-the-art technologies for energy efficiency in 5G was presented. The authors addressed areas such as energy efficiency at the BS level, with 5G NR; caching; and using SDN, energy-efficient NOMA, energy-efficient resource sharing, and interference-aware energy efficiency.

A few reviews considered energy-efficient resource management in cellular networks and other communication systems, such as [9,10,11,25]. In [8], the state-of-the-art techniques for energy-efficient wireless networks are introduced, including EE metric, network deployment strategies, resource management, relay, cooperative communication, and multi-input multi-output (MIMO) and OFDM technologies. Meanwhile, in [7], the authors reviewed sleep mode techniques to reduce power and energy consumption in base stations, with the objective of developing a green cellular network. Thereby, the authors highlighted the importance of green mobile networks. In [11], the authors presented a survey on the research activities on radio resource management in machine-to-machine (M2M) communications for LTE/LTE-A cellular networks. Here, they considered access control, radio resource allocation, and power management. They also reviewed radio resource management for M2M communications in heterogeneous networks in conjunction with recent standard activities and continuing research issues. Furthermore, the authors in [25] presented a survey of the state-of-the-art caching techniques developed in different types of cellular networks, such as macro-cellular networks, heterogeneous networks, device-to-device networks, C-RAN, and F-RAN, by comparing different algorithms in terms of performance metrics including throughput, backhaul cost, power consumption, and network delay.

Although substantial efforts have been undertaken to review the resource management in different wireless network environments and clouds, none of these specifically cover the resource management approaches proposed for C-RAN in recent years. A few articles presented a description of this architecture, wherein they mentioned the benefits and drawbacks of C-RAN for 5G cellular systems [19,23]. However, computational and radio resource management are not specifically considered in these articles. Radio resource management is discussed in the literature for C-RAN, H-CRAN, and other communication scenarios [7,8,11,22], but computational resource management is not covered in these surveys. Different techniques of base stations have also been reviewed in [9,12,13]. However, they do not explicitly include the recent techniques for RRH clustering, which is reviewed in this survey.

### 1.2. Contributions of This Survey

To the best of the authors’ knowledge, no recent survey exists that explicitly and dedicatedly describes the resource management techniques for C-RAN, proposed in recent years. This survey paper seeks to provide a holistic perspective of resource management techniques for C-RAN. Specifically, it focuses on the recently proposed (i.e., from 2016) resource management mechanisms categorized in terms of radio resource management (RRM) and computational resource management (CRM) techniques. The aim of this study is to fill the research gaps in the literature. The contributions of this paper are summarized below:

- A concise review of the existing surveys related to the topic of our interest.
- The evolution of C-RAN architecture and the components of a C-RAN are overviewed so that new researchers can gain a fundamental understanding of this architecture. The advantages of C-RAN are discussed, and a short description of Heterogeneous C-RAN and Fog RAN are presented by mentioning the challenges in C-RAN.
- A comprehensive survey of the resource management techniques for C-RAN is provided by categorizing the techniques into CRM and RRM techniques. The CRM techniques cover the RRH clustering techniques in C-RAN, which includes location-aware, load-aware, interference-aware, QoS-aware, and throughput-aware RRH clustering. The RRM techniques include power control, joint optimization, and sum-rate optimization techniques.
- The problem formulation and techniques used in the reviewed papers and the goal of the papers are presented together with a description of each approach. The evaluation techniques and performance metrics used in the reviewed approaches are also discussed separately by presenting a comparison among all the schemes. This allows for a better comprehension of the validation techniques considered in C-RAN resource management techniques.
- We highlight the challenges and future research directions in C-RAN resource management, such as user mobility, QoS and QoE requirements, dynamic traffic load, demand forecasting, and NB-IoT.

### 1.3. Paper Organization

**Figure 1:**
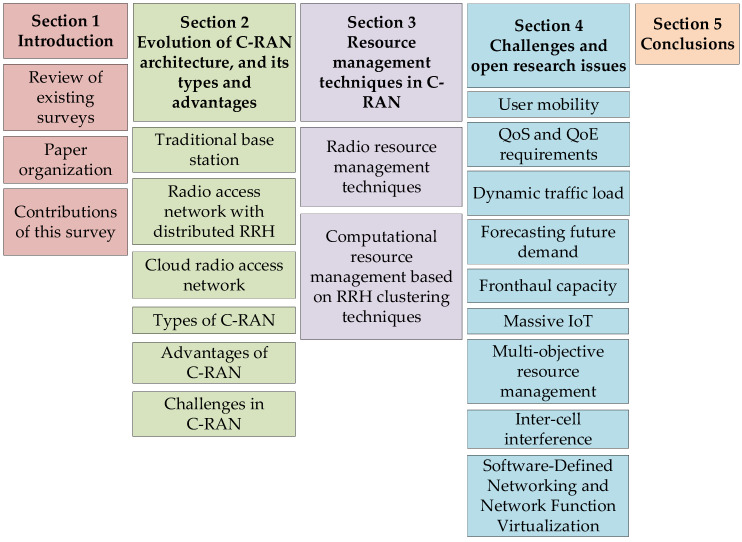
Organization of this survey.

The paper is organized as illustrated in Figure 1. In Section 2, the evolution of the C-RAN architecture, components of a C-RAN together with the advantages of this architecture, and the challenges in C-RAN are presented. In Section 3, a comprehensive survey on the state-of-the-art resource management techniques for C-RAN is described. Section 4 briefly describes the challenges and continuing research issues in the resource management of C-RANs. Finally, the paper is concluded with a brief summary in Section 5.

## 2. Evolution of C-RAN Architecture, and Its Types, Advantages, and Challenges

C-RAN is a mobile network architecture where baseband resources are pooled in a centralized and virtualized manner so that the resources can be shared among different base stations. This section provides an overview of the evolution of this architecture from the traditional base station through RAN with distributed RRH. Different types of C-RAN architecture described in the literature, the advantages of this architecture, and a short introduction of the H-CRAN architecture is also presented in this section.

### 2.1. Traditional Base Station

In cellular networks, the base station is an essential component that facilitates the communication between users and the networks under whose coverage they are located. The base station has two functionalities named as baseband processing and radio functions. The functions of the baseband processing unit include coding, modulation, and Fast Fourier transform (FFT). The radio unit is responsible for digital processing, frequency filtering, and power amplification [5]. In the traditional architecture, both baseband processing and radio functions are carried out inside a base station. The base station is situated at the base of a tower and connected to the antenna located at the top of the tower using coaxial cable. Different base stations are connected to each other through the X2 interface. Each base station is connected with the mobile core network through the S1 interface. This architecture is being used since the 2G mobile network.

### 2.2. Radio Access Network with Distributed RRH

**Figure 2:**
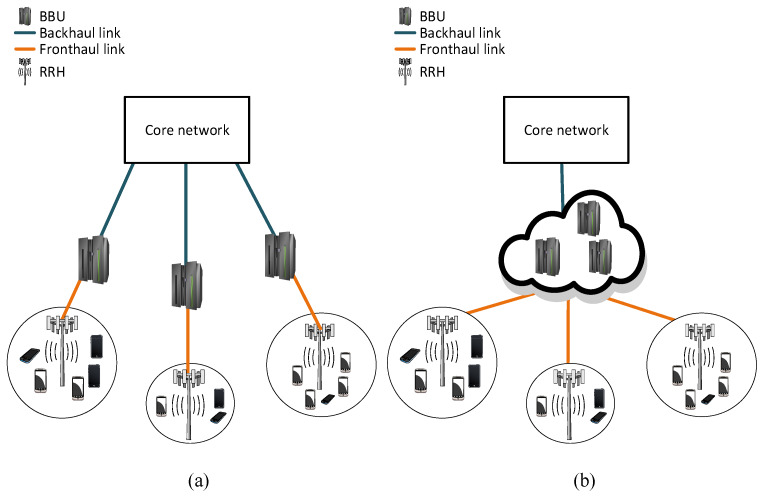
(**a**) Architecture of RAN with distributed RRH, (**b**) Architecture of cloud RAN.

In the distributed RAN architecture, the base station is separated into two units called BBU and RRH. The RRH is placed on the top of a tower with the antenna, and the BBU is placed in a convenient location that is easily accessible from the RRH. These two units are interconnected by a common public radio interface (CPRI), also known as fronthaul link, as shown in Figure 2a. The RRH functions as a transceiver for the mobile users, and the BBU processes calls and forwards traffic to the mobile core network via carrier Ethernet backhaul, also called backhaul link. The RRH provides the interface to the fiber and carries out digital processing, digital to analog conversion, analog to digital conversion, power amplification, and filtering [26]. The distance between the BBU and RRH can be extended up to 40 km, although this would result in processing limitation and propagation delay [5]. This RAN architecture is developed for 3G and 4G mobile networks.

### 2.3. Cloud Radio Access Network

In a C-RAN, the BBUs are relocated from the individual cell sites to a centralized and virtualized BBU pool. A C-RAN is composed of three main components: BBU pool, RRH, and fronthaul link (Figure 2b). C-RAN architectures proposed by both industries and academia are described in [27]. This subsection presents a brief overview of the components of a C-RAN.

#### 2.3.1. BBU Pool

The BBU pool is a centralized, shared, and virtualized site functioning as a data processing center. Individual units, i.e., virtual BBUs, can be stacked together without direct linkage or interconnected to allocate resources based on the dynamic user demand on the connected RRHs. Each BBU pool can support multiple RRHs. In the BBU pool, multiple BBUs are interconnected via the X2 interface, which is highly cost-effective and yields improved performance. The S1 interface connects a BBU to the mobile core network, also called backhaul link. BBUs are composed of high-performance programmable processors and apply real-time virtualization technology. From the implementation perspective, BBUs are installed on virtual machines (VMs) using a hypervisor over physical computing cores present in the cloud data center [28]. Further details on the virtualization of BBUs are provided in [4,5,28,29,30,31].

#### 2.3.2. Remote Radio Head

The RRHs are distributed over a geographical area where mobile users are provided with communication services, which is referred to as service area or cell sites. The area around an RRH where transmission conditions are favorable enough to maintain a connection between a user and an RRH according to the required QoS is called the coverage area of the RRH. The RRH has two parts: a transmitting part and a receiving part. In the transmitting part, a digital signal is received via a CPRI interface, converted to analog, upconverted to an RF frequency, amplified, filtered, and then output via an antenna. The receiving part receives a signal from the antenna, filters it, amplifies it, down-converts it to an IF Frequency, and then converts it to a digital signal before sending it out via the CPRI to a fiber for further processing.

#### 2.3.3. Fronthaul Link

Fronthaul is the connection layer between a BBU and a set of RRHs. It provides high bandwidth and low latency links to handle the requirements of multiple RRHs. This link can be setup using different technologies including optical fiber communication, cellular communication, and millimeter-wave (mmWave) communication [29]. Although optical fibers provide the highest bandwidth requirement, they are highly expensive and not flexible for deployment. In the case of cellular or mmWave communication, they are comparatively less expensive and more convenient for implementation. However, these do not provide the required bandwidth as an optical fiber connection and also cause more latency. The deployment of a C-RAN with cost-optimal fronthaul is discussed in [32].

### 2.4. Types of C-RAN

Two approaches for splitting the base station functionalities between BBU and RRH within a C-RAN are presented by China Mobile in [4]. Based on these, C-RANs can be categorized into two types: fully centralized and partially centralized.

#### 2.4.1. Fully Centralized

**Figure 3:**
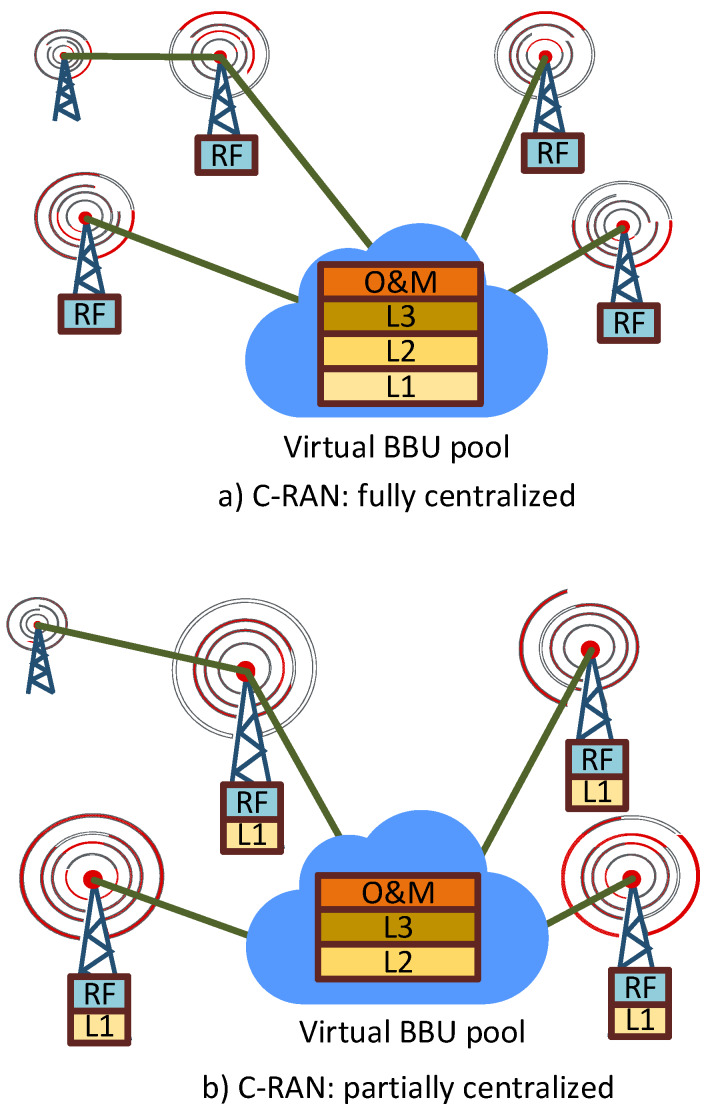
Types of C-RAN: (**a**) fully centralized C-RAN, (**b**) partially centralized C-RAN.

In this architecture, as shown in Figure 3a, layer 1 functionalities such as sampling, modulation, resource block mapping, antenna mapping, and quantization; layer 2 functionalities such as transport-media access control; and layer 3 functionalities such as radio resource control are located in the BBU [23]. It has the capability of supporting the multi-standard operation, expanding network coverage area, maximizing resource sharing, and providing multi-cell collaborative signal processing. Notwithstanding the significant benefits of this architecture, the load on the fronthaul link is substantial and the bandwidth requirement is very high.

#### 2.4.2. Partially Centralized

In this architecture, as shown in Figure 3b, the RRH performs the radio functions as well as the functions related to layer 1. Meanwhile, the functions of the higher layers, layer 2 and layer 3, are still executed in the BBU. As the baseband processing is shifted to the RRH from the BBU, the bandwidth requirements between the BBU and RRH become lower in a partially centralized C-RAN. However, it also has certain drawbacks: as the baseband processing is integrated with the RRH, it is less flexible for upgrade and less convenient in terms of multi-cell collaborative signal processing [4].

However, the authors in [29] extended this classification to three categories by adding another architecture for C-RAN: hybrid centralized architecture. In this architecture, a part of the layer 1 functions is performed in the RRH, whereas the others are performed in the BBU. The RRH performs the user- or cell-specific functions related to the signal processing. This architecture displays higher flexibility for resource sharing and the facility for reducing the energy consumption and communication overhead of a BBU

### 2.5. Advantages of C-RAN

This subsection presents a brief description of the advantages of C-RAN architecture: adapting dynamic traffic, load balancing, convenience of operation and maintenance, cost reduction, and interference minimization. 

#### 2.5.1. Adapting to Dynamic Traffic Load

Mobile phone users perform dynamic movement throughout the day. This results in varying traffic loads in different areas at different times of the day. At certain times of the day, a few BSs are oversubscribed, whereas others are in the idle mode. Consequently, radio resources are utilized inefficiently, and a large amount of processing power is wasted. After the centralization of the BBUs into the BBU pool, the RRHs with low traffic loads can be merged and managed by one BBU, whereas the RRHs with high traffic loads can be assigned to multiple BBUs according to the user demand. This can improve the overall utilization rate and ensure the effective utilization of the computing resources, which results in a significant statistical multiplexing gain. It has been demonstrated in [33] that a flexible, reconfigurable mapping between a BBU and an RRH following different traffic profiles maximizes the statistical multiplexing gain. 

#### 2.5.2. Load Balancing

In a C-RAN, load balancing can be performed on the BBU as well as RRH sides. This is because BBUs from a large area are co-located in the same BBU pool [5]. Load balancing can be performed on the BBU side by assigning appropriate BBU resources within a pool. Load balancing can be achieved on the RRH side according to the capacity of the BBUs within a BBU pool, when users move from one cell to another. C-RAN provides the facility to switch off a BBU or an RRH depending on the traffic load in the network. BBU can be switched off by an appropriate association of BBU–RRH. When the traffic load in the RRH is low, the associated BBU can be switched off by connecting that RRH to another BBU within that BBU pool. The RRH can also be turned on/off dynamically according to the user demand on the cell site and by associating the users with another RRH.

#### 2.5.3. Convenience of Operation and Maintenance

The centralized architecture of a C-RAN makes it scalable and simplifies its upgrade and maintenance. Although the number of RRHs in C-RAN architecture may not be decreased, its functionality becomes more convenient as the size of the tower reduces, and RRH can sit on poles with minimum site support and management. To increase the coverage of a network, MNOs only have to install an RRH on the cell site and associate a BBU with that RRH in the cloud data center. C-RAN provides the facility to install virtual resources on the cloud depending on the user demand. Moreover, the maintenance of an RRH becomes more convenient in C-RAN. In addition, as BBUs are centralized in a cloud, in addition to cost reduction, the management of the BBU pool becomes simpler compared to that for a traditional RAN.

#### 2.5.4. Cost Reduction

MNOs deploy more base stations to process the ever-increasing amount of mobile traffic. This results in higher network power consumption, eventually resulting in higher cost. The centralization of the BBU pool enables the efficient utilization of BBUs and reduces the CAPEX for deploying base stations in different areas and the OPEX for the operation, maintenance, and upgrade of the base stations. The electricity cost required for a traditional RAN can also be reduced by deploying a C-RAN because BBUs are absent in the cell site. Moreover, a few BBUs can be switched off during a period of low traffic load, which would result in power savings. A C-RAN also reduces the requirement of cooling resources, which accounts for 46% of the power consumption of cell sites in the traditional RAN [4]. 

#### 2.5.5. Interference Minimization

In the traditional RAN, the movement of users from one cell to another, which alters the coverage area of the associated base stations, causes interference. This, in turn, results in decreased QoS. In the C-RAN architecture, many RRHs from different cell sites are connected to BBUs sharing the same BBU pool. User dynamicity does not cause interference as the change in coverage area of RRHs by users does. This is because BBUs from a large area are co-located to the same BBU pool, and the RRHs connected to those BBUs are also attached to that BBU pool. This mitigates the interference caused by user movement between different cell sites.

### 2.6. Challenges in C-RAN

There are two major challenges in C-RAN architecture, namely the fronthaul capacity issue and complications of virtualization techniques [34]. The practical fronthaul is often capacity, and time-delay constrained that significantly reduces spectral efficiency and energy efficiency gains [35]. The use of virtualization techniques in cellular networks like C-RAN is far more complicated due to the unique features of wireless communication. Two RAN architectures (Heterogeneous C-RAN and Fog RAN), for compensating the challenges in C-RAN, are proposed and evaluated in the literature. This section provides a brief discussion of these two architectures.

#### 2.6.1. Heterogeneous C-RAN

H-CRAN is a novel paradigm that combines a heterogeneous cellular network architecture with the cloud infrastructure. In a heterogeneous network, various classes of low power nodes (LPNs) such as pico base stations, femto base stations, and small cell base stations are distributed throughout the network. The LPN is one of the key components for increasing the cellular network capacity in dense areas with a high traffic demand. It can be combined with a high-power node (HPN) such as a macro or micro base station to form a heterogeneous network [7]. HPNs are generally employed for increasing network coverage and controlling network signals to prevent unnecessary handovers in small cells. The full advantages of both heterogeneous network and C-RAN have been utilized in an H-CRAN, by incorporating HPNs into CRAN, in which multiple heterogeneous networks can be converged to provide seamless coverage [36]. This results in improved spectral and energy efficiencies as well as increased data rates. In [23], the authors presented a few similarities and dissimilarities between C-RAN and H-CRAN. According to this study, both architectures have a large number of RRHs connected to the BBU pool to achieve a high cooperative gain and to increase the energy efficiency. Moreover, the functional splits of both the architectures include higher layer functions that perform in a BBU and radio functions that perform in RRHs. However, an H-CRAN has simplified capacity and time delay in fronthaul links. The requirements of the fronthaul are alleviated with the participation of HPNs, as control signaling, and system broadcasting data are transmitted to users through HPNs [36]. Moreover, in H-CRAN, an RRH can be turned off for improving the energy efficiency during low traffic demand, whereas the BBU pool is responsible for managing the RRHs that are in the sleeping mode. The authors in [37] studied subchannel allocation and power optimization in NOMA-based Heterogeneous networks, considering energy harvesting and cross-tier interference mitigation. Energy harvesting is utilized due to the limited available energy at the base station nodes. They considered simultaneous wireless information and power transfer for achieving the efficient utilization of energy resources and ensure the QoS simultaneously. 

#### 2.6.2. Fog RAN

F-RAN is a promising paradigm that enhances the C-RAN architecture by allowing the RRHs to be equipped with local caches for storing frequently requested contents and signal processing capabilities. F-RAN takes the benefit of both fog computing and C-RAN, which extends the traditional cloud computing to the network edge. The main idea is to ensure that all traffic is not offloaded directly from the centralized cloud server, while some local traffic should be delivered from the caching of adjacent RRHs or smart UEs [35]. This collaborative processing minimizes the overload of the constrained fronthaul links as well as alleviates the queuing and transmission delay. The optimal design of an F-RAN requires edge caching, fronthaul, and wireless transmission to be jointly optimized to leverage the edge processing and virtualization. The authors in [38] presented an information-theoretic model for F-RAN, aiming at characterizing the key tradeoff between the system performance in terms of delivery latency and resources available for fronthaul, caching, and wireless transmission. In the F-RAN architecture, UEs access F-RAN adaptively, and the transmission mode is selected based on UEs movement speed, distance, location, QoS requirements, processing and caching capabilities. More details on four different transmission modes in F-RAN and also the comparison on the characteristics of the three promising architectures (C-RAN, H-CRAN, and F-RAN) are described in [35].

## 3. Resource Management Techniques in C-RAN

**Figure 4:**
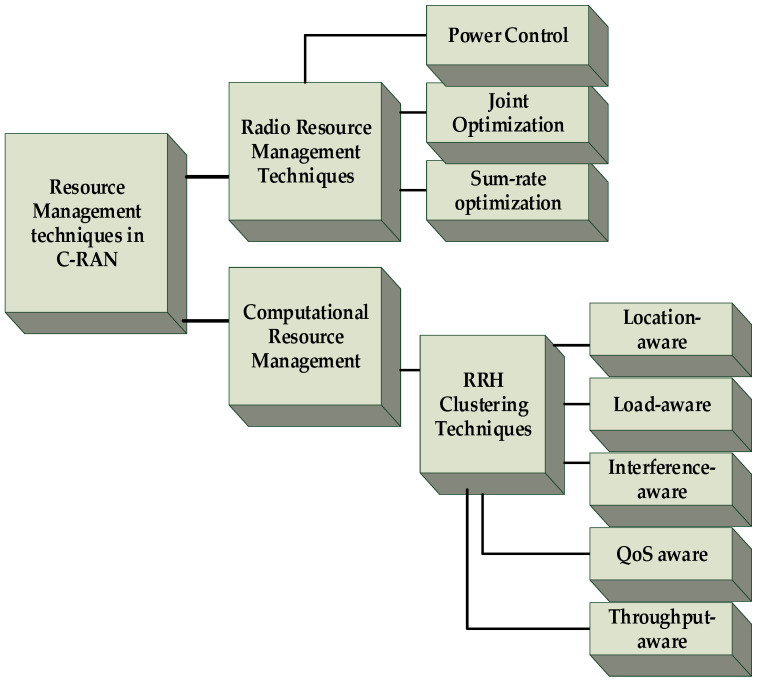
Resource management techniques in C-RAN.

There are two types of resources in C-RAN, similar to other cellular network architectures: computational resources and radio resources. The computational resources, which are required in the base stations, include memory, processing power, data storage, time, and bandwidth. Computational resource management can be performed by managing the resources in the BBU pool, i.e., by managing the BBU resources. In this study, we focus on different RRH clustering techniques for BBU resource management (also called BBU–RRH association techniques) by switching on/off BBUs to allocate computational resources optimally according to the user demand. In cellular communication, radio resource implies radio frequency spectrum, which is highly limited. RRM focuses on effectively utilizing the limited radio frequency spectrum resources and radio infrastructure. It includes strategies and algorithms for transmit power control, dynamic channel allocation, spectrum management, cache management, and joint optimization. The resource management techniques reviewed in this paper are categorized, as shown in Figure 4, into RRM and CRM. The RRM techniques are classified into power control schemes, joint optimization schemes, and sum-rate optimization. For CRM, we considered the RRH clustering techniques. These are categorized into location-aware, load-aware, interference-aware, QoS-aware, and throughput-aware techniques.

### 3.1. Radio Resource Management Techniques

Many techniques have been proposed by different researchers for radio resource management C-RAN by optimizing different metrics and following various constraints to enhance QoS, increase energy efficiency, and reduce cost. In this section, the state-of-the-art RRM techniques (categorized into power control schemes, joint optimization schemes, and sum-rate optimization) are reviewed considering the main objectives of the work, problem formulation, and techniques used in the work. Moreover, the evaluation techniques used in the reviewed works and the performance metrics considered for evaluating the schemes are discussed separately in Section 3.1.2.

#### 3.1.1. Power Control Schemes

Xu et al. proposed a two-step deep reinforcement learning (DRL)-based framework for dynamic allocation of resources in C-RAN, to minimize the total power consumption while satisfying the user demand in the network [39]. The authors defined the states space, action space, and reward function for the DRL agent and proposed a two-stage DRL technique: an offline deep neural network (DNN) construction phase and an online dynamic deep Q learning phase. First, the DRL agent determines the active set of RRHs by turning itself on or off, to reduce the action space size. It then determines an optimal resource allocation solution (beamforming) with the active set of RRHs by solving a convex optimization problem.

Zhao et al. investigated the power and bandwidth allocation problem in C-RAN with the aim of minimizing the total transmission power for a specified set of RRHs while satisfying the average rate and spectral efficiency requirement [40]. The problem was formulated as a traffic density-based RRH selection task with minimum power consumption. A series of network constraints were considered, such as transmission power budget, spectrum limitation, traffic demand, and spectral efficiency requirement. To solve this problem, they developed an efficient local search algorithm based on the optimal values of the power and bandwidth allocation problem and introduced three local improvement operations (“add,” “open,” and “close”) to determine locally optimal solutions rapidly. 

In [41], the work of [40] is extended. The authors designed a static RRH selection scheme for load balancing among RRHs. Furthermore, based on this, a dynamic RRH switching mechanism considering a fairness index to measure the imbalance degree of traffic load is presented. The RRH selection is performed using an efficient local search procedure in conjunction with a user association algorithm, considering the bandwidth and power budgets of the RRH and energy consumption of the system. They designed a bandwidth and power allocation algorithm for determining the minimum power consumption of the RRH while satisfying the rate requirements of users. Subsequently, the fairness index was defined by introducing three local improvement operations: “open,” “close,” and “exchange.” The RRH switching mechanism is designed such that when the bandwidth and power requirements of the user are not satisfied and the fairness index is larger than a predefined threshold, the switching is triggered for load rebalancing while the signaling overhead of the system is controlled. However, the selection of the fairness index threshold is critical because a signaling overhead may be generated and decrease the system performance because of the handover of users, during RRH switching. 

Aldaeabool et al. [42] proposed a strategy for switching a BBU on/off according to the traffic load in the associated RRH by using a host server (HS) in the BBU pool, which assigns a newly arrived user to a certain BBU. They formulated an optimization problem of reducing the number of BBUs with low loads by transferring the load to neighboring BBUs with the available capacity. They proposed a combined bin packing method (BPM) and modified the best fit decreasing (MFBD) algorithm to solve this problem. Here, each BBU is considered as a bin for traffic load balancing.

In [43], Lee et al. investigated the power consumption tradeoff between BBUs and RRHs. They formulated a theoretical model for BBU aggregation to determine the optimal traffic threshold for RRH switching to minimize the total power consumption. The authors considered an architecture involving (1) subareas, with three types of RRHs, based on the coverage area of RRHs and (2) two modes for the subareas (LC mode and SC mode) based on the states and types of RRHs in that area. First, the expected number of LC mode and SC mode RRHs are calculated. Next, the traffic loads carried by the active RRHs are calculated. Then, the expected number of active BBUs is derived from the BBU aggregation model, which is based on the BPM. Subsequently, the expected total power consumption is determined from the expected number of active or sleeping RRHs and BBUs and their power consumption. Finally, the authors presented a procedure based on the bisection method for determining the optimal threshold. 

#### 3.1.2. Joint Optimization Schemes

In [44], Lyazidi et al. proposed DRAC, a two-stage design for dynamic resource allocation in a C-RAN in conjunction with real-time BBU-RRH assignment, considering the constraints in transmission power and Signal-to-interference-plus-noise-ratio (SINR) for user equipment (UE). In the first stage, they formulated the resource allocation problem as a mixed linear integer problem (MILP). They then solved it using the branch and cut algorithm (BCA), which associates the best spectrum set of frequency/time resources dynamically to incoming UEs. In the second stage, the optimal number of BBUs required and RRHs to be turned on to handle the traffic load is calculated. This problem was formulated as a multiple knapsack problem (MKP). Here, the RRHs are the objects, and the BBU capacity to handle the real-time traffic load is the knapsack. It was solved by IBM’s linear solver CPLEX, which determined the optimal result using highly marginal computation time. 

Lyazidi et al. investigated joint downlink resource allocation and admission control for mobile users in an orthogonal frequency division multiple access (OFDMA)-based C-RAN as an optimization problem [45]. The objective was to determine the optimal PRB allocation to maximize the total user throughput in the system while considering the QoS and mobile user data rate requirements, maximum transmission power, and fronthaul link limitation constraints. They proposed a two-stage resource allocation and admission control (RAAC) algorithm. The resource allocation problem was first solved using the fixed time branch-and-cut algorithm without the data rate constraints. Then, the fast admission control algorithm was utilized based on the output of the former problem to select the largest set of mobile users to be accepted in the system, considering the dropped data rate constraint. 

Wang et al. proposed a C-RAN structure with a mobile cloud (virtual machine) co-located in the BBU pool with BBUs, to investigate the joint energy minimization and resource allocation. The objective was to enhance both the performance and energy efficiency of the C-RAN in [46]. The architecture is such that the mobile cloud executes the computationally intensive task, whereas the BBUs are responsible for transferring the executed results to the RRH for sending it to the UE. The joint energy minimization is formulated as a non-convex optimization problem, which is further reformulated into an equivalent convex problem, based on power minimization and sum data rate maximization that is transformed into a minimization of weighted sum mean square error (MSE) problem. The objective is to minimize the energy cost of the mobile cloud and network considering the QoS, i.e., the time constraints. A WMMSE-based iterative algorithm is proposed. It can solve the joint resource allocation between the mobile cloud and C-RAN and thereby improve the system performance and energy saving. 

#### 3.1.3. Sum-rate Optimization

In [47], Liao et al. investigated the impact of available computing resources on the PHY transmission characteristics by formulating a user–RRH association problem for uplink transmission in a C-RAN. The main objective was to maximize the network sum-rate under limited computing resources. At first, the binary integer non-linear programming problem was transformed to a general non-linear programming problem for reducing complexity. Then, an iterative sub-optimal algorithm was proposed to solve the problem by updating the objective function iteratively until the optimal solution is achieved.

Table 2 presents the research activities mentioned in this paper on RRM techniques in C-RANs. The application, goal, problem formulation, and techniques that are used in the relevant works are mentioned.

#### 3.1.4. Evaluation Techniques for Radio Resource Management Methods

Different evaluation techniques and performance comparisons based on different metrics for the RRM techniques in C-RANs have been proposed by researchers. Table 3 presents the evaluation techniques together with the performance metrics for the RRM schemes reviewed in this work (which are mentioned in Table 2).

The performance of the proposed two-step DRL-based approach in [39] is evaluated by comparing it with two widely used baselines: single BS association (SA) and full coordinated association (FA). It is illustrated that the approach yields better results in terms of the total power consumption and user demand in each decision time. In this work, the reduction in the action space size and state transition overheads (power consumption during a transition from active/sleep to sleep/active) via a two-step DRL-based framework was considered. Zhao et al. used simulations to compare the efficient local search algorithm as proposed in [40] with two other methods (no RRH selection and greedy-based RRH selection) in terms of the power consumption during each iteration with different numbers of RRHs as well as TDAs and with different spectral efficiency. It was demonstrated that the power consumption for different spectral efficiency requirements is almost equivalent. This indicates that the spectral efficiency is not a significant factor in the power consumption of the C-RAN. However, in [41], which is an extension of [40], the performance of the proposed method is evaluated through simulation and compared with the SINR-based scheme, cell range expansion, and the Min-power scheme, in terms of the number of satisfied users, active RRH for current users, and outrage probability for different call departure rates. The authors in [42] evaluated the performance of the proposed MBFD through simulation. They demonstrated that the MBFD algorithm yields higher performance than BFD and traditional networks in minimizing the number of active BBUs and the power consumption for normalized traffic load in C-RAN. This is because MBFD achieves load balancing while saving energy, after modification from BFD. A simulation is presented and analyzed in terms of the traffic threshold and total power consumption of the network, for evaluating the performance of the method proposed in [43]. It was concluded that the consideration of only the power consumption of RRHs results in increased total power consumption of the network, because of the BBUs. Notwithstanding the marginal difference between the theoretical and simulation results, the proposed scheme reduces the total power consumption while optimizing BBU aggregation.

In [44], the proposed DRAC algorithm was simulated in MATLAB, and the performance of the approach was compared with those of a few state-of-the-art schemes to demonstrate the high throughput satisfaction rate, minimal power consumption, and significant reduction in the number of BBUs required with the maximum number of RRHs handled per BBU. The DRAC scheme performs a good tradeoff between the satisfaction rate and overall power consumption for both the SINR threshold levels. In [45], the performance of the proposed algorithm RAAC was evaluated by means of simulation in a wireless LTE C-RAN environment with 19 hexagonal RRHs and three users uniformly distributed in each cell. Then, it was compared with a semi-definite positive relaxation-based algorithm (SDPRA) and a fast greedy algorithm (FGA) in terms of the number of admitted users, total transmission power, and number of BBUs. It was demonstrated that it increases user admission by 9% and 11%, respectively. Moreover, it saves 23% and 53%, respectively, more transmission power. In [46], the proposed approach was simulated using MATLAB with CVX tool, considering five mobile clones co-located with the BBUs in the BBU pool. The performance of the joint scheme was compared with the individual energy minimization schemes in terms of the total energy consumption for varying QoS requirements and CPU cycles of the task. The proposed method outperformed the separate solutions in all cases.

In [47], simulations were conducted by varying the computing resource constraints. The effect of the computing resources in both user–RRH association schemes and achievable system sum-rate was depicted. The users could not obtain high data rates even if they had high SINR when the computing resource was scarce. However, the optimal user association scheme can be achieved with sufficient computing resources. Moreover, a knee point was presented. It indicated the computing resources required by a BBU pool for achieving a satisfactory transmission rate in a C-RAN network.

#### 3.1.5. Lessons Learned

The minimization of the number of BBUs required to handle traffic loads with a reduced number of RRHs resulted in an improved network capacity as well as less overhead in fronthaul links of the network. It also reduced the inter-cell interference. For improving the energy efficiency of a CRAN, RRHs need to be selectively turned on/off such that they cause less overhead in the transport network and reduce the number of active BBUs within the BBU Pool. However, the RRH switching method results in load balancing among all the RRHs and minimum power consumption of the system. Thereby, a stable scenario can be achieved for addressing unexpected future traffic demands effectively.

Power consumption is maximum during the peak traffic load because of the usage of a larger number of BBUs, which results in more energy consumption. Therefore, it is more effective to consider the dynamic traffic load for maintaining the QoS requirements as well as energy efficiency, while designing algorithms for minimizing power consumption.

### 3.2. Computational Resource Management Based on RRH Clustering Techniques

**Figure 5:**
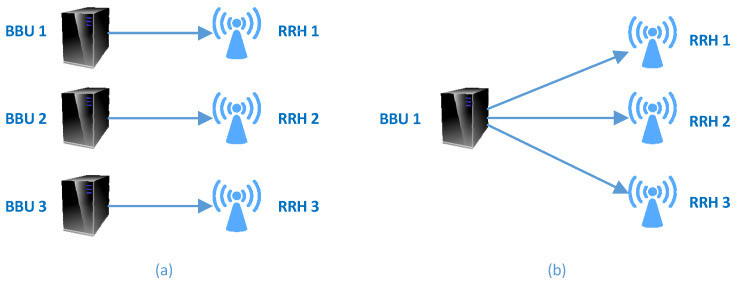
BBU-RRH mapping: (**a**) one-to-one mapping (**b**) one-to-many mapping.

In conventional RAN architectures, a one-to-one logical mapping exists between RRHs. This implies that one BBU is assigned to one RRH. However, in a C-RAN architecture, it is feasible to establish a one-to-many mapping by assigning one BBU to many RRHs. The former results in the deployment of small cells wherein a user connects to one BBU through one RRH. In this mapping, the BBUs in the BBU pool are not utilized efficiently because all the RRHs do not have equal traffic load perpetually. In a one-to-many mapping, a user can connect to the same BBU through multiple RRHs because multiple RRHs are assigned to the same BBU. This is similar to a distributed antenna system (DAS). The mappings are depicted in Figure 5. The computational resource usage of the BBUs in a C-RAN is minimized via assigning multiple RRHs to one BBU by clustering RRHs according to the user rate requirements. Limiting the number of active BBUs decreases the power consumption and increases the radio resource utilization because commonly grouped RRHs share the same BBU resource. Deciding which RRHs must be grouped together by forming a cluster to reduce the number of active BBUs is known as an RRH clustering problem. Many techniques have been proposed for formulating and solving the RRH clustering problem in C-RANs. In [48], the authors formulated the RRH clustering problem as a modified bin packing problem to reduce the number of active BBUs while maintaining the QoS. 

#### 3.2.1. Location-Aware RRH Clustering

Karneyenka et al. proposed a two-step resource management mechanism for minimizing expensive handovers within the BBU pool while improving the QoS [49]. First, they designed a hierarchical location-based clustering algorithm to cluster VBSs according to the location of their associated RRHs. This strategy divides the entire area in a grid and places RRHs close to each other in a cluster. It accepts the clustering distance and the entire RRH list as inputs and produces the clustered RRH as the output. Then, they proposed a packing algorithm, which simultaneously addresses host overutilization and improves cluster packing using location, mobility, and handover information. The complexity analysis of the algorithms and performance comparison with other existing schemes in terms of the QoS (number of expensive inter-cluster handover) and resource (RRH, hosts, and energy) consumption was demonstrated. 

#### 3.2.2. Load-aware RRH Clustering

Mishra et al. formulated the RRH clustering problem as a generalization of the classical bin packing optimization problem. Here, BBUs are considered as bins, and RRHs are the item sets to be packed [50]. They designed DRA, a dynamic RRH assignment algorithm for offloading one or more RRHs from an overloaded BBU to a less loaded BBU having computing resources available to serve the incoming RRH(s), thereby forming a new cluster. DRA reduces the number of active BBUs by 87% in comparison with one-to-one BBU-RRH mapping and consumes 25% of the time required by First-fit Decreasing (FFD) for the urban cellular deployment of 1000 RRHs.

In [51], Taleb et al. formulated the RRH clustering problem as a coalition formation game. The objective was to maximize the network performance by balancing the network throughput, handover frequency, and power consumption. They proposed a centralized approach based on an exhaustive search and a distributed approach based on the merge-and-split rule to form clusters, following either the utilitarian order (which considers the global network utility) or the Pareto order (which considers individual RRH utility). They defined the utility functions considering the network performance (specifically, the throughput and handover frequency) as well as the network power consumption at the cloud side. In the centralized approach, an exhaustive search algorithm explores all the feasible partitions and selects the best one according to either the Pareto order or the utilitarian order. This search becomes intractable because the number of partitions increases rapidly. Therefore, a distributed approach is adopted to overcome this complexity.

The author of [52] and [48] formulated a joint optimization problem in [53] for minimizing the power consumption and handover rate of UEs. The author solved it via a hybrid algorithm consisting of two stages. Unlike the previous works, this algorithm requires less amount of signaling load information. In the first stage, a central controller predefines the number of active BBUs. In addition, all the RRHs compete (based on a potential game among themselves) to select the most adequate BBU with the lowest cost, until a Nash equilibrium is attained. The second stage is based on a centralized scheme to activate or deactivate a BBU based on the throughput per cluster, and the number of users served per cluster when a BBU is deactivated. This method reduces the signaling load over the fronthaul link between BBUs and RRHs because RRHs are not required to send their load and radio conditions to the BBUs.

Hesham et al. formulated the RRH clustering problem to minimize the number of activated BBUs based on the current state of the user and dynamic resource requirement [54]. A modified k-means clustering technique and two heuristic solutions are proposed to form the RRH clusters. In k-means, the input is the matrix of resource blocks. Here, a row represents an RRH, and each RRH is clustered based on the pattern of resource blocks that it provides to each user. This algorithm is modified such that an overloaded BBU or cluster migrates its users to the least overloaded cluster, and the migrated users who have the minimum difference in the reserved resource blocks between its current cluster and the migrated cluster are selected. The two heuristic solutions (where one takes the next maximum, and the other takes the subsequent minimum) create RRH clusters in two steps. This is because both require user assignment to an RRH before it is assigned to a BBU prior to the clustering, to achieve efficient load balancing. In addition, this study proposed the concept of developing an independent mini distributed antenna system in an indoor C-RAN for 4G networks, to increase user rate provisioning with fewer resource blocks as well as to minimize the number of BBUs.

In [55], Chen et al. proposed a deep learning-based framework in C-RAN optimization to maximize the capacity utilization and minimize the deployment cost to achieve statistical multiplexing gain. The framework is two-phased: (1) dynamic RRH profiling phase consisting of RRH traffic forecasting and RRH complementarity measurement and (2) complementary RRH clustering phase consisting of weighted-graph-based RRH modeling and distance-constrained RRH clustering. In the first phase, a multivariate long short-term memory (MuLSTM) is utilized for traffic pattern forecasting of RRHs in a future period based on historical traffic data. Furthermore, the complementarity of RRHs is measured based on the traffic snapshot forecast. In the second phase, a weighted graph model is constructed to represent the relationship of RRHs. In addition, a distance-constrained complementarity-aware (DCCA) algorithm is proposed to cluster RRHs iteratively such that the complementarity among RRHs is increased within each cluster and reduced across different clusters.

In [56], Yu et al. considered the selection of active RRUs and the allocation of multiple computational resources in BBUs. The objective was to minimize the number of active BBUs required to serve all the users in the network. They formulated the multi-resource allocation problem as a multi-dimensional bin packing BP problem by first considering that the virtual machines (VMs) in BBUs have diverse resource requirements, and then considering each BBU as a bin and each VM as an item. In this problem, the number and sizes of items can be adjusted for packing by varying the associations between the RRU and UEs. The size of the BBU indicates its resource capacities. They proposed an iterative resource allocating (IRA) algorithm to solve this problem. It assigns a VM to the BBU with the required resource allocation by following the largest-dot-product-first strategy. This strategy prevents over allocation of computational resources and achieves a higher packing efficiency than the first-fit strategy. However, while assigning VMs to BBUs, the VM’s requirements of all the resource types need to be considered.

#### 3.2.3. Interference-Aware RRH Clustering

In [52], Boulos et al. presented an interference-aware clustering algorithm (IACA) for RRH clustering with the objective of minimizing the network power consumption, subject to a minimum throughput requirement. They designed the system model to perform the clustering within a few hours and divided the area into discrete zones using a square meshing method. The problem was formulated as a set partitioning problem and solved using a greedy heuristic solution with complexity reduction. The objective was to determine the RRHs that minimize the power consumption in accordance with the throughput constraints. The results of the heuristic revealed performances very close to the optimal solution and with less complexity. Although power saving is achieved using this approach, the energy efficiency was lower than that with the bin packing approach. Moreover, the RRH clustering strategy is designed considering a full buffer traffic model that does not take the traffic dynamicity into account.

The work of [52] is extended in [48]. Here, the objective was to minimize the power consumption and to minimize the RRH re-association rate while forming a new cluster. This is because the re-association of RRHs with different BBUs may cause handover for the users connected to the RRH. Unlike the previous work, this work presented a two-stage heuristic solution. Here, the first stage decided the RRH re-association with a BBU when the minimum throughput requirements per user are not fulfilled. The second stage addressed the minimization of the number of active BBUs by moving RRHs to the BBU with a moderate throughput per user. A tradeoff between the power saving and re-association rate was illustrated via numerical results. That is, when the algorithm focused more on power saving, the re-associate rate increased, and vice versa. 

Extending [51], the authors formulated a joint user association and RRH clustering problem in [57] considering inter-cluster interference. They divided it further into two sub-problems to reduce complexity. The main goal was to maximize the network throughput and minimize the network power consumption. The RRH clustering problem was solved by an iterative, low complexity heuristic algorithm based on merge-and-split rules, in which the RRHs combined and organized themselves into independent clusters such that the network utility was maximized. This process was repeated until convergence, which implies that no more RRH clustering was required. 

#### 3.2.4. QoS-aware RRH Clustering

In [58], the authors investigated the load-balancing problem in a C-RAN. The objective was to reduce the number of blocked calls and maximize the QoS. A key performance indicator (KPI) was used to represent the QoS. It was defined as the number of blocked calls. The scenario was developed such that 19 RRHs were distributed randomly and connected to a BBU pool consisting of two BBUs with three sectors each. The particle swarm optimization (PSO) algorithm was used for BBU–RRH mapping. Herein, the best particles were represented by a vector result, where each particle characterized a feasible combination of RRHs distributed in the sectors of the BBUs. 

In [59], Yao et al. formulated a QoS-aware joint BBU–RRH mapping and user association problem in a C-RAN. The objective was to minimize the system cost by minimizing the power consumption of the RRHs and the number of virtual BBUs (VBs). The joint optimization problem was decomposed into two subproblems to reduce computing complexity. Accordingly, the user association problem was solved first, and the optimal solution was utilized to solve the BBU–RRH mapping problem. The former problem was solved using a Lagrangian relaxation algorithm, and the latter was solved by the best fit decreasing algorithm (LAGA-BFD). The BBU–RRH mapping problem was formulated as a bin packing problem. Here, VBs were backpacks, and the RRHs were the objects to be placed in the backpacks. To solve this problem, the BFD was utilized such that one RRH will be connected to one VB, which would be the best fit for it. This implies that the VB has the minimum remaining capacity after the addition of the RRH, and a VB was added when there was no VB available to accommodate the RRH.

Khan et al. investigated the dynamic RRH allocation considering a self-optimized C-RAN with the objective of maximizing the network QoS by traffic load balancing and minimizing handovers in the network [60]. The problem was formulated as an integer-based optimization problem in a self-organizing network (SON) by defining multiple KPIs. Based on this, a SON controller residing inside the BBU pool can identify the network configuration and perform dynamic RRH allocation as well as load balancing in the network. The KPIs considered here were for blocked users and handovers. Here, inter-BBU, intra-BBU, and forced handovers were considered. The self-organized C-RAN (SOCRAN) algorithm utilizing two evolutionary algorithms (genetic algorithm (GA) and deterministic particle swarm optimization (DPSO)) was proposed for solving the problem. It was demonstrated by numerical analysis that both the GA and DPSO provide optimal performance for small networks and nearly optimal performance for large networks, whereas DPSO outperformed GA in different network scenarios.

In [61], the self-optimized architecture for C-RAN (SOCRAN) proposed in [60] was utilized for performing semi-static cell differentiation and integration (CDI) and dynamic BBU–RRH mapping for load balancing simultaneously. CDI was investigated with the objective of utilizing the network resources effectively while maintaining the overall network QoS. Herein, a cell was split into multiple small cells and vice-versa, in response to the measured load information in one or more cells in the network. A two-stage design was proposed, where the first stage computes the optimal number of BBUs according to load demand and activates or deactivates RRHs based on the CDI concept for handling traffic load. The second stage was modeled as an integer-based linear optimization problem for effective BBU–RRH mapping, to maximize QoS while minimizing handovers for network load balancing. Furthermore, this problem was solved using a DPSO algorithm. Three KPIs were considered for BBU–RRH mapping, taking into account the load fairness index, network throughput, and handovers.

The authors in [62] proposed a self-optimized algorithm for dynamic RRH clustering in C-RAN based on load prediction. Meanwhile, the traffic load in each cell was predicted using the Markov model, and the optimal solution for BBU–RRH mapping was determined using the GA. The objective was to maximize the QoS by minimizing the connection blocking and handover failure with a balanced load. The QoS was determined by two KPIs, which were represented as the inverse of the blocked calls in the network and handovers. During the prediction phase, the Markov model considered the location of the user and developed groups of cells by acquiring the neighbor cells to form a Markov state, based on the user behavior. This was performed to reduce complexity. After predicting the number of users in each cell, the GA was executed in advance according to the predicted load to determine the optimal BBU–RRH mapping and obtain the appropriate configuration for minimizing the number of blocked users with the minimum execution time delay. The QoS criteria of the existing mapping were analyzed and compared with the predicted mapping. The new BBU–RRH mapping was attained if the number of predicted users and the number of users in that time match.

#### 3.2.5. Throughput-Aware RRH Clustering

Salhab et al. investigated a throughput-aware RRH clustering problem with the objective of maximizing the throughput for end-users by maintaining multiple constraints on BBU resources [63]. A two-stage approach was proposed. Here, the throughput value and requirements of each RHH were calculated first considering the SINR values and the distance of RRH and users. Then, a k-dimensional multiple-choice knapsack problem (k-MCKP) was formulated considering the calculated result as inputs. Unlike previous works, this work considers multiple BBUs with several constraints, based on real hardware implementation. The arrival of UEs was modeled by a Poisson point process, in which UEs were spread and connected to RRHs. They proposed a heuristic to solve the k-MCKP, which was simple and efficient, and obtain values close to the optimal solution.

#### 3.2.6. Evaluation Techniques for RRH Clustering Methods

Different evaluation techniques and performance comparisons based on different metrics for RRH clustering techniques have been proposed by researchers. Table 4 presents the evaluation techniques and the performance metrics for the RRH clustering methods reviewed in this work (listed in Table 5).

In [49], a complexity analysis of the proposed location-aware VBS clustering algorithm and location and mobility-aware packing algorithm, and a comparison of their performances with those of other existing schemes in terms of QoS (number of expensive inter-cluster handover) and resource (RRH, hosts, and energy) consumption were described. In [52], the performance of IACA was compared with that of the classical bin packing algorithm through simulation. In addition, the heuristic solution was compared with the optimal solution in terms of the number of active BBUs, energy efficiency, power saving, and mean throughput per user. However, in [48], which is an extension of [52], whereas the performance evaluation was performed similarly, the performance metrics considered were power saving, re-association rate, and mean throughput per user. The authors in [51] compared the performance of the optimal centralized approach (as centralized Pareto or centralized utilitarian, named based on the approach) with those of the grand coalition and the no-clustering method through simulation. The comparison was in terms of the number of active BBUs, throughput, power consumption, and handover for different traffic load scenarios. Furthermore, the performances of the distributed and centralized approaches were compared using the Pareto and utilitarian order, with respect to the number of active BBUs, utility function, and throughput. It was demonstrated that the distributed approaches achieve performance very close to those of the centralized approaches in all the cases. 

The performance of the algorithm in [57] was compared with the optimal exhaustive search-based solution, no-clustering solution (in which one BBU is exclusively dedicated to one RRH), and grand coalition (in which all the RRHs are connected to one BBU). However, in [53], the performance of the algorithm was evaluated by comparing it with a centralized algorithm and bin-packing algorithm, which displays a good tradeoff between power saving and re-association rate. In [54], the modified k-means technique and the two heuristic algorithms were simulated and tested on MATLAB and compared in terms of the number of activated BBUs and the number of clusters and resource blocks. K-means performed well in terms of the number of clusters and tightness of the clusters, although it required a longer time to attain convergence. In [58], through simulation, the performance of the proposed algorithm was compared with a solution proposed in the literature [60], having an identical scenario and another optimal solution that divides the users between BBU sectors without considering their distributions in the RRHs. The load balancing of BBUs considering a different number of users was considered as the performance metric. It was demonstrated that the PSO could converge faster with fewer iterations than those for the solution provided in the literature, and optimize the QoS by reasonably balancing the BBU–RRH sectors. 

In [59], the LAGA-BFD algorithm for solving the joint optimization of user association and BBU–RRH mapping problem was simulated. Furthermore, the performance was evaluated with different numbers of RRHs (6–14), a different number of UEs, and different traffic arrival rates, considering more and less stringent QoS requirements. The impact of all the above parameters on the system cost was compared while comparing the proposed method with the optimal ILP by using CPLEX and the nearest-first scheme. In [63], the proposed heuristic for solving the k-MCKP-based clustering technique was simulated via Monte Carlo simulation in MATLAB. Then, the performance was compared with the optimal solution and two no-clustering approaches (no-clustering upper bound and no-clustering lower bound) in terms of the end-user throughput, spectral efficiency, and execution time. However, in [60], for analyzing and verifying the performance of the proposed algorithm for RRH allocation (SOCRAN), three problem scenarios are presented by varying the numbers of RRHs and BBUs, and the sector served by the BBUs. The algorithm was tested 30 times over 30 configurations for each benchmark. The average of the results obtained was considered for Monte Carlo analysis. The performances of both the GA and DPSO in the SOCRAN algorithm were compared with those of exhaustive search and the k-means clustering algorithm in terms of the QoS, the number of blocked users, and the number of handovers, via simulation. In [61], the performance of DPSO was analyzed in two problem scenarios considering small and large networks and compared with those of the GA and ES algorithm. All the algorithms were executed 50 times with 50 configurations for both the problem scenarios. The average of the results obtained was considered for Monte Carlo analysis. Moreover, the CDI-based CRAN concept was also compared with a fixed-CRAN scenario through simulation.

In [55], a real-world mobile network traffic dataset was collected for evaluating the performance of the proposed framework. Furthermore, a set of 61 daily traffic snapshots was generated based on that data. The snapshots of the first 70% were used for generating the training data. The remaining 30% of snapshots were used for the test data. Then, the MuLSTM model with two stacked LSTM layers was constructed and trained using TensorFlow, thereby ensuring that the network learns the potential temporal and spatial structure. Then, the proposed method was compared with the traditional approach as well as the ARIMA-DCCA, WANN-DCCA, and MuLSTM-DC methods, in terms of the traffic forecast error, average capacity utility, and overall deployment cost. In [62], for simulation, the network scenario was developed considering 19 RRHs grouped into six sectors with two BBUs. Then, the algorithm was executed to determine the optimal BBU–RRH mapping by predicting the number of users at that time. In addition, the performance of the GA is compared with that of ES in terms of the QoS with varying numbers of iterations. Here, the number of blocked connections in the network is minimized. The authors in [56] considered a network with densely deployed RRUs at distances of 100 m, and for BBU, they considered CPU, memory, and disk as the computational resources for simulation. The performance of the iterative resource allocating (IRA) algorithm in solving the multi-resource allocation problem was evaluated by comparing it with main resource packing, no UE aggregation, two-stage optimization, and exhaustive search, in terms of the number of active BBUs, with different numbers of RRHs.

#### 3.2.7. Takeaway Points

The assignment of multiple RRHs to a BBU enhances the transmission power density received by the user. This minimizes the number of resource blocks required to satisfy user demand. Because each BBU can support a larger number of users through one-to-many mapping of BBU–RRH, the number of active BBUs required for providing the demand required in the network is decreased. Moreover, RRH clustering results in reduced interference among the RRHs. This, in turn, results in an increased throughput in the case of exploited interference. This is referred to as inter-cell interference cancellation. 

Frequent re-association between BBUs and RRHs owing to RRH clustering results in the degradation of the QoS of the network. This is because handovers occur when users become connected to different BBUs. Therefore, a few authors considered the network QoS while solving the RRH clustering problem.

## 4. Challenges and Open Research Issues

This section discusses the challenges of efficient resource management in C-RANs and future direction for the researchers of this field. It is highly crucial to resolving a few of these issues for the advancement of mobile networks.

### 4.1. User Mobility

User mobility has a remarkable impact on resource management in C-RANs. Understanding user mobility is highly important for resource optimization and algorithm evaluation in mobile network planning and handover mechanisms [64]. The virtualized BBUs in a C-RAN require resources based on the mobility of the users in the network. Therefore, the disregard of user mobility in the problem while managing resources may result in an inefficient solution. A few mobility-aware resource management mechanisms have been proposed for C-RANs. In [49], a location and mobility-aware clustering and packing algorithm were designed for virtual BBU clustering and placement, to minimize resource consumption.

In the future, researchers can design a user mobility-aware clustering algorithm for BBU or RRH clustering in a C-RAN from a load balancing perspective. Moreover, machine learning techniques can be applied for user mobility prediction such that resource allocation can be performed according to the prediction.

### 4.2. QoS and QoE Requirements

Many researchers have worked with QoS-aware resource management problems in C-RAN in a different manner. One of the potential methods to solve the problem can be the use of deep learning (DL) techniques. In [65], DL was applied to solve the QoS-aware power management problem because of its efficiency of mapping inputs to outputs. The fundamental concept was to train a deep neural network to obtain the optimal power management results by learning the potential relationship between the input variables automatically. The same concept can be implemented for a C-RAN by training neural networks to obtain optimal resource management.

Whereas QoS is related to the network services, the consideration of parameters such as throughput, latency, jitter, and quality of experience (QoE) is another important subjective measurement that addresses the satisfaction level of a user while using a telecommunication service. Considering the forthcoming requirements of the 5G cellular network, it is also necessary to develop QoE-driven resource management schemes and identify appropriate KPIs for the network. The knowledge of the instantaneous and average QoE per user may aid the MNOs in modifying the network resource usage for each cell size, set different bandwidth limits dynamically, and identify the minimum resource required to achieve the desired QoE [66]. Developing efficient cache management can be a good approach to improving QoE by reducing the transmit delay in the network. 

### 4.3. Dynamic Traffic Load

The traffic load in a cellular network varies across areas and hours of the day. When resources are not allocated based on the dynamicity of the traffic load, a few base stations are overloaded and others underused. This results in inefficient resource management. Therefore, it is essential to design a resource management mechanism considering this issue. Most of the works that have been performed on traffic-aware resource management provided solutions based on the static traffic load rather than the dynamic traffic environment.

A feasible solution could be the design of algorithms for traffic prediction such that resources can be allocated accordingly. Deep learning techniques can be applied in that case. The authors in [67,68,69] employed DL techniques for mobile traffic prediction. Here, they achieved significantly higher accuracy than conventional approaches by extracting spatio-temporal features.

### 4.4. Forecasting Future Demand

Demand forecasting is a key issue in C-RAN resource management. Efficient forecasting techniques can yield optimal resource allocation strategies to minimize energy consumption. Machine learning techniques can be an appropriate solution for this problem, particularly the utilization of a neural network-based predictor for forecasting the future demands in the network. Another feasible method could be the adoption of reinforcement learning (RL) techniques, where the RL agent would learn the network environment and forecast the future demand and allocate resources based on that learning. The RL agent would allocate resources to the BBUs associated with those RRHs by learning from the user demands of different RRHs in a BBU pool.

### 4.5. Fronthaul Capacity

The fronthaul link between a BBU and RRHs substantially impacts the performance of a C-RAN. This link needs to have a high capacity and low latency. The fronthaul capacity must be considered while allocating BBU resources to the RRHs through RRH clustering during the design of the clustering algorithm. The mechanisms have to be designed such that the load on the fronthaul links does not exceed the capacity of the links. Fronthaul-aware approaches must be considered during the formulation of the optimization problem, for designing energy-efficient solutions while maintaining the QoS requirements. Moreover, other architectures such as H-CRAN and F-RAN, which are evolved from C-RAN, can be considered for future research as they can be utilized to solve the high load problem in fronthaul link. The authors in [70] studied the delivery phase of an F-RAN for an arbitrary pre-fetching, with the goal of maximizing the delivery rate while satisfying fronthaul capacity and per-RRH power constraints. They took into account two basic fronthauling modes, namely hard transfer and soft transfer, and also a hybrid mode. In the soft transfer mode, C-RAN principle is followed for the fronthaul links for transferring quantized baseband signals, whereas, in the other mode, the requested files that are not in the local caches are transmitted by the fronthaul links. Another attractive technology is narrowband Internet of things that requires moderate fronthaul capacity as it can operate in a narrow bandwidth and can be further investigated for implementation in C-RAN environment [71].

### 4.6. Narrowband IoT

IoT will be a crucial part of future cellular networks. It is estimated that 212 billion IoT devices will be connected in the near future. Therefore, the aggregated traffic from these devices would account for almost half of all internet traffic [72]. Few studies have investigated C-RAN resource management-integrating IoT devices. A new RAN technology introduced by 3GPP, called narrowband Internet of Things (NB-IoT), is implemented on a flexible software-defined radio-based C-RAN in [71]. NB-IoT has the benefits of less complexity in transceiver design, low power consumption, reduced cost for the radio chip, and increased coverage. Relaxed latency requirements along with simplified baseband processing complexity make NB-IoT attractive for C-RAN platform. However, there exist some challenges for this technology that requires further investigation, and it can be a potential research area for future researchers. Another work presented in [73] utilized C-RAN architecture in ambient backscatter communication, to jointly cope with direct-link interference suppression and imperfect channel estimation. In this architecture, secondary edge nodes give network access to ambient backscatter passive and semi-passive sensors with communication capabilities, and the channel estimation and direct-link interference suppression are managed by the cloud processor. 

Although these studies were able to manage the IoT support in cellular networks from the perspective of centralized IoT transmission coding/decoding, further investigation is needed considering IoT optimized control procedure. In addition, as RRHs are densely deployed in C-RAN network, they may become congested when a small number of RRHs are overburdened with IoT devices. Moreover, the mobility of IoT devices would deteriorate the situation [74]. It is essential to develop techniques to support massive connections as well as ensure low-latency and highly reliable data transmission. To address these issues, an H-CRAN can be developed as an effective solution in conjunction with edge computing capability. Low latency can be provided by shifting data analysis and decision making to the network edge.

### 4.7. Multi-Objective Resource Management 

With the convergence of information and communication technology, the available types of resources in C-RANs would comprise computational resources, network resources, radio resources, and cache resources. Most of the ongoing researches has considered only one type of resource in the C-RAN architecture while developing resource management schemes. Optimizing multiple resources would simultaneously facilitate the achievement of higher system performance in this network. The system model for linking various types of resources needs to be explored to simplify the simultaneous optimization. A significant challenge for multi-dimensional resource management would be the handling of multiple resources that are allocated at different time scales. This is known as a two-timescale problem [75]. This challenge needs to be addressed in the future for efficient multi-objective resource allocation. 

### 4.8. Inter-Cell Interference

The C-RAN incorporates robust computing capability and large data storage, which requires proper resource allocation and interference mitigation. Notwithstanding the design of a near-optimal algorithm for resource allocation, the performance may be significantly sub-optimal and the measurement of inter-cell interference tedious, time-consuming, and highly inaccurate, owing to the incorrect modeling of interference among different cells [76]. It is necessary to design an algorithm that solves this dilemma so that the optimization of resource management is achieved in conjunction with interference mitigation with appropriate measurement and reduced cost. This task can be challenging because, over time, the interference estimated would be dynamic. Therefore, multiple measurements would be required to ensure the accuracy of the measurements within each connection. The authors in [77] investigated the resource management in a NOMA based F-RAN, focusing on the power and sub-channel allocation considering the co-channel interference. The problem is designed as a non-cooperative game, and many-to-many two-sided matching algorithm is studied for sub-channel allocation. 

### 4.9. Software-Defined Networking and Network Function Virtualization

Software-defined networking (SDN) makes a separation between control and data planes, in which network switches are considered as dummy packet forwarding devices logically controlled by a centralized entity [5]. Network management, security policy deployment, scaling up or down, and troubleshooting are some of the major advantages of this network paradigm compared to traditional networking system [78]. In C-RAN architecture, SDN can be a suitable solution for dynamic resource allocation, balancing traffic load among BBUs and automatic recovery in case of hardware failure [5]. However, the data forwarding flow in SDN is mainly at the IP layer, and combining the functions of medium access control and the physical layer for C-RAN is a challenging task. For proper rollout of C-RAN with SDN, this issue challenge needs to be overcome.

Network Function Virtualization (NFV) enables the transferring of network functionalities from dedicated hardware to software-based applications, which can be shared in a flexible and dynamic way [5]. It is essential to design the technique to virtualize the SDN controller to run on the cloud server, which can be migrated to a location according to network needs. Implementation of a real-time processing algorithm, virtualization of the BBU pool for C-RAN, and dynamic signal processing for dynamic traffic load are challenging, and researchers are still working to find the optimal virtualization technique for C-RAN.

## 5. Conclusion

Several techniques proposed for efficient resource management in C-RAN are analyzed and discussed in this paper. This study explicitly presents an in-depth comparison among resource management techniques proposed recently for C-RAN architecture, whereas previous surveys focused on a few particular issues in resource management in different wireless networking scenarios or base stations. Considering the continuous increasing traffic demand, it is highly necessary for researchers to determine solutions for managing resources efficiently while maintaining the QoS requirements and minimizing power and energy consumption. The selection of appropriate performance metrics is also a crucial task. Therefore, this study focused on the performance metrics used in different works, as well as the evaluation techniques performed. We have also presented the current and future challenges in C-RAN as well as future research directions for solving the challenges effectively.

## Figures and Tables

**Table 1:** Existing surveys on related topics.

Year of Publication	Ref.	Topic(s) of Survey	Resource Management	C-RAN
2013	[10]	Energy-efficient wireless communications	✓	✕
2014	[14]	Resource management in clouds	✓	✕
2014	[22]	Radio resource management for heterogeneous LTE/LTE-A networks	✓	✕
2014	[17]	Resource allocation and monitoring in cloud computing	✓	✕
2015	[7]	Resource sharing in heterogeneous cloud radio access networks	✓	✓
2015	[18]	Machine learning applications for energy-efficient resource management in cloud computing environments	✓	✕
2015	[9]	Energy-efficient base-stations sleep-mode techniques in green cellular networks	✓	✕
2015	[15]	Evolutionary computation for resource management of processing in cloud computing	✓	✕
2016	[8]	Resource management toward 5G RANs	✓	✓
2016	[16]	Resource scheduling in cloud computing	✓	✕
2016	[12]	Strategies for switching off base stations in heterogeneous networks for greener 5G systems	✓	✓
2017	[11]	Radio resource management in machine-to-machine communications	✓	✕
2017	[20]	Energy efficiency on fully cloudified mobile networks	✓	✕
2018	[6]	Resource allocation and scheduling methods in cloud	✓	✕
2018	[13]	Clustering techniques for RRH in 5G networks	✓	✕
2018	[25]	Caching techniques in cellular networks	✓	✕
2018	[21]	Recent advancements of heterogeneous radio access networks	✕	✓
2019	[19]	Cloud radio access network for 5G cellular systems	✕	✓
2019	[23]	RAN architectures for 5G mobile communication system	✕	✓
2019	[24]	Recent trends and open issues in energy efficiency of 5G	✓	✕
	**Our survey**	**Resource management in cloud radio access networks**	✓	✓

**Table 2:** Radio resource management techniques in C-RAN.

Strategy	Ref.	Application	Goal	Problem Formulation	Technique Used
Power Control	[39]	Power-efficient resource allocation	Minimize total power consumption by determining an optimal beamforming solution	Second order cone optimization problem	DRL
	[40]	RRH selection based on traffic density	Reduce total power consumption	Mixed integer programming problem (MIPP)	Efficient local search algorithm (ELSA)
	[41]	Static RRH selection and dynamic RRH switching	Load balancing among RRHs and controlling the signaling overhead of the system	MIPP	ELSA and adaptive trigger mechanism
	[42]	Switching BBU on/off based on traffic load	Reduce number of active BBUs and power consumption	Linear integer programming	Combined BPM and MFBD
	[43]	Threshold-based RRH switching and BBU aggregation	Minimize power consumption in both BBU and RRH	Bin packing problem	Bisection method to determine the optimal threshold
Joint Optimization	[44]	Downlink physical resource block allocation and BBU–RRH assignment	Minimize number of BBUs required to handle traffic load	Mixed linear integer problem and multiple knapsack problem	BCA
	[45]	Downlink resource allocation and admission control	Determine the optimal PRB allocation for maximizing the total user throughput	MILP	Fixed time BCA
	[46]	Joint energy minimization and resource allocation	Minimize energy cost in mobile cloud and network considering QoS	Convex optimization problem	WMMSE-based iterative model
Sum-rate Optimization	[47]	User–RRH association for uplink transmission	Maximize network sum-rate under limited computing resources	Non-linear programming problem	Iterative sub-optimal algorithm

**Table 3:** Performance evaluation techniques and performance metrics used in radio resource management.

Ref.	Evaluation Technique	Performance Metrics
[39]	Performance comparison with Single BS association and Full coordinated association	Total power consumption and user demand
[40]	Comparison with No RRH selection and greedy-based RRH selection	Power consumption with different numbers of RRHs, TDAs and spectral efficiency
[41]	Comparison with SINR-based scheme, cell range expansion, and the Min-power scheme	Number of satisfied users and active RRH, outrage probability
[42]	Comparison with BFD and traditional networks	Number of active BBUs and power consumption
[43]	Simulation of the theoretical analysis	Optimal traffic threshold, total power consumption
[44]	Performance comparison with QP-FCRA, Iterative GSB algorithm and Semi-static, adaptive switching	Throughput satisfaction rate, spectrum spatial reuse, transmitted power, number of BBUs and RRHs required
[45]	Simulation and performance comparison with SDPRA and FGA	Number of admitted user, total transmission power, number of BBUs
[46]	Simulation and performance comparison with separate energy minimization solution	Total energy consumption
[47]	Simulation showing the impact of changing computing resources	User–RRH association strategy, achievable sum rate

**Table 4:** Performance evaluation techniques and performance metrics used in RRH clustering technique.

Ref.	Evaluation Techniques	Performance Metrics
[49]	Simulation and performance comparison with other existing C-RAN schemes	QoS (number of expensive inter-cluster handover) and resource (RRH, hosts, and energy) consumption
[52]	Simulation and comparison with classical bin packing algorithm and comparison of heuristic solution with optimal solution	Number of active BBUs, energy efficiency, power-saving, and mean throughput per user
[48]	Simulation and comparison of heuristic solution with optimal solution	Power saving, re-association rate of users, and mean throughput per user
[50]	Simulation and comparison with FDD bin packing algorithm	Computational resource gain and power saving
[57]	Simulation and comparison with the optimal exhaustive search-based solution, no-clustering solution, and grand coalition	Number of active BBUs, user interference, user throughput, power consumption, and network utility
[53]	Simulation and comparison with a centralized algorithm and bin-packing algorithm	Power saving, re-association rate of users, mean throughput per user, and execution time
[51]	Simulation and performance comparison with grand coalition and no-clustering method	Number of active BBUs, throughput, power consumption, handover
[54]	Simulation and performance comparison among three proposed techniques	Number of active BBUs, number of clusters and resource blocks
[58]	Simulation and performance comparison with literature model and the optimal approach	BBU load balancing with number of users
[59]	Simulation and performance comparison with optimal ILP by CPLEX and nearest-first scheme	System costs for different numbers of RRHs, UEs, and average arrival rate
[63]	Simulation and performance comparison with the optimal solution and no-clustering scheme	End-users throughput, spectral efficiency, and execution time
[60]	Simulation and performance comparison with ES and k-means clustering	QoS, blocked users, and handovers
[61]	Simulation and performance comparison of DPSO with GA and ES, and CDI-CRAN with F-CRAN	QoS, load fairness index, network throughput, and handover
[55]	Training the DL model and performance comparison of the test set with traditional, ARIMA-DCCA, WANN-DCCA and MuLSTM-DC methods	Traffic forecast error, average capacity utility, and overall deployment cost
[62]	Simulation and performance comparison of GA with ES	Number of blocked connections, QoS
[56]	Simulation and performance comparison with Main Resource Packing, No UE Aggregation, Two-stage Optimization and ES	Number of active BBUs

**Table 5:** Comparison of RRH clustering techniques.

Strategy	Ref.	Optimization Objective	Goal	Problem Formulation	Technique Used
Location-aware	[49]	Energy	Reduce resource consumption through virtual BBU clustering and placement	N/A	Location-aware VBS clustering algorithm and location and mobility-aware packing algorithm
Load-aware	[50]	Power	Minimize power consumption and the number of active BBUs	Classical bin packing optimization problem	Lightweight, load-aware dynamic RRH association algorithm
	[51]	Throughput, power, handover	Maximize network performance by balancing network throughput, handover frequency and power consumption	Coalition formation game	Centralized approach based on exhaustive search and distributed approach based on merge and split rule
	[53]	Power	Minimize power consumption and handover rate of UEs simultaneously	Joint optimization problem	Two-stage hybrid algorithm
	[54]	Computational usage	Minimize the number of activated BBUs to reduce computational usage	Modified K-means-based clustering and two heuristic algorithms	N/A
	[55]	Distance span	Maximize the capacity utilization and minimize the deployment cost	Community detection problem	Multivariate LSTM for forecasting and Distance-constrained complementarity-aware algorithm for clustering
	[56]	Computational usage	Minimize the number of active BBUs required to satisfy VM resource demand	Multi-dimensional bin packing problem	Iterative resource allocating algorithm
Interference- aware	[52]	Power	Reduce network power consumption with minimum throughput requirements	Set partitioning problem	Interference-aware clustering algorithm
	[48]	Power	Reduce power consumption and BBU–RRH re-association rate	Tunable bi-objective optimization problem	Exhaustive search and two-stage heuristic solution
	[57]	Throughput, power	Maximize network throughput and minimize network power consumption	Mixed integer non-linear programming problem	Low complexity heuristic algorithm based on merge-and-split rules
QoS-aware	[58]	Blocked calls	Minimize the number of blocked calls and load balancing between BBUs	N/A	Particle swarm optimization algorithm
	[59]	System cost	Minimize power consumption of RRHs and number of virtual BBUs	Integer Linear Programming problem, Bin packing problem	LAGA-BFD
	[60]	Blocked user, handover	Maximize network QoS by traffic load balancing and minimize handovers	Integer-based optimization problem	GA and DPSO
	[61]	fairness index, throughput, handover	Maximize QoS and minimize handovers for network load balancing	Integer-based liner optimization problem	CDI algorithm and DPSO
	[62]	blocked calls, handover	Maximize the QoS by minimizing the connection blocking and handover failure	Markov decision process	Markov model for prediction and GA for optimization
Throughput-aware	[63]	throughput	Maximize the system throughput for end-users	k-dimensional multiple-choice Knapsack problem	Simple and efficient heuristic algorithm

## Abbreviations

The following abbreviations are used in this manuscript:

BBU	Base band unit
BCA	Branch and cut algorithm
BPM	Bin packing method
CAPEX	Capital expenditures
CDI	Cell differentiation and integration
CPRI	Common public radio interface
C-RAN	Cloud radio access network
CRM	Computational resource management
DAS	Distributed antenna system
DCCA	Distance constrained complementarity-aware
DL	Deep learning
DNN	Deep neural network
DPSO	Deterministic particle swarm optimization
DRL	Deep reinforcement learning
ELSA	Efficient local search algorithm
FA	Full coordinated association
FFD	First-fit decreasing
FFT	Fast Fourier transform
FGA	Fast greedy algorithm
F-RAN	Fog radio access network
GA	Genetic algorithm
H-CRAN	Heterogeneous cloud radio access network
HPN	High power node
HS	Host server
IACA	Interference-aware clustering algorithm
IoT	Internet of things
IRA	Iterative resource allocating
k-MCPC	k-dimensional multiple-choice knapsack problem
KPI	Key performance indicator
LAGA-BFD	Lagrangian relaxation algorithm and best fit decreasing
LPN	Low power node
M2M	Machine to machine
MFBD	Modified best fit decreasing
MILP	Mixed linear integer problem
MIMO	Multi-input multi-output
MIPP	Mixed integer programming problem
MKP	Multiple knapsack problem
mmWave	millimeter wave
MNO	Mobile network operator
MSE	Mean square error
MuLSTM	Multivariate long short-term memory
NB-IoT	Narrowband internet of things
NFV	Network function virtualization
OFDMA	Orthogonal frequency division multiple access
OPEX	Operational expenditures
PSO	Particle swarm optimization
QoE	Quality of experience
QoS	Quality of service
RAAC	Resource allocation and admission control
RAN	Radio access network
RF	Radio frequency
RL	Reinforcement learning
RRH	Remote radio head
RRM	Radio resource management
SA	Single BS association
SDN	Software-defined networking
SDPRA	Semi-definite positive relaxation-based algorithm
SINR	Signal-to-interference-plus-noise ratio
SOCRAN	Self-organized C-RAN
SON	Self-organizing network
UE	User equipment
VB	Virtual BBU
VM	Virtual machine
